# The Effect of Relaxation Therapy on Hypoxia During Intravenous Propofol Anesthesia in Patients With Pre-operative Anxiety: A Prospective Randomized Controlled Trial

**DOI:** 10.3389/fmed.2022.797337

**Published:** 2022-03-15

**Authors:** Yiling Fang, Qi Jing, Silu Cao, Xiaoru Sun, Hui Zhang, Li Tian, Cheng Li

**Affiliations:** ^1^Department of Anesthesiology and Perioperative Medicine, Shanghai Fourth People's Hospital, School of Medicine, Tongji University, Shanghai, China; ^2^Translational Research Institute of Brain and Brain-Like Intelligence, Shanghai Fourth People's Hospital, School of Medicine, Tongji University, Shanghai, China; ^3^Clinical Research Center for Anesthesiology and Perioperative Medicine, Tongji University, Shanghai, China; ^4^Department of Anesthesiology, Shanghai Tenth People's Hospital, Tongji University School of Medicine, Shanghai, China; ^5^Department of General Practice, Zhangjiagang First People's Hospital, Affiliated to Soochow University School of Medicine, Suzhou, China

**Keywords:** intraoperative hypoxia, pre-operative anxiety, propofol anesthesia, relaxation therapy, induced abortion

## Abstract

**Background:**

This study aimed to investigate the effect of relaxation therapy on hypoxia during intravenous propofol anesthesia in patients with pre-operative anxiety.

**Methods:**

Two-hundred and eighty patients were randomly categorized in the experimental group (relaxation therapy group) and control group. The Spielberger State-Trait Anxiety Inventory (S-STAI) was administered 30 to 60 min pre-operatively to assess the patient's current anxiety status and select appropriate patients. Patients in the experimental group received pre-surgical relaxation therapy. Decrease in oxygen saturation during the procedure was recorded for each patient group, and the relevant data were compared between the two groups.

**Results:**

The basic S-STAI scores of the experimental and control groups were 56.88 ± 2.91 and 57.27 ± 3.56, respectively (*p* = 0.331). The difference was not statistically significant. The incidence of hypoxia in the experimental group during painless artificial abortion [routine blood oxygen saturation (SpO_2_) <95%, duration >15 s] decreased from 30 to 12.3%.

**Conclusion:**

Relaxation therapy may effectively reduce the incidence of hypoxia during painless artificial abortion by using less dose of propofol. It may help patients relieve their anxiety and improve perioperative safety.

**Trial Registration:**

Chinese Clinical Trial Registry (ChiCTR2000032109).

## Introduction

Pre-operative anxiety is a psychological reaction before surgery. It manifests as discomfort, apprehension, and fear (1). The three main reasons for pre-operative anxiety are unknown surgery, disease, and the possibility of death (2). It is understandable that patients have mild anxiety before and after surgery. However, severe anxiety often causes intraoperative hemodynamic problems (3) and affects the progress of rehabilitation (4–7). The incidence of pre-operative anxiety in adult patients can be as high as 80% (8). High pre-operative anxiety independently predicts increased post-operative mortality and the incidence of serious complications (9). Moreover, anxiety is an independent risk factor for intraoperative hypoxia (10). Individuals under the age of 30 years and women are more likely to have higher levels of anxiety (11–13). Hence, young women were asked to participate in this study. Patients who underwent painless artificial abortion in this study were at a high risk of pre-operative anxiety. Anti-anxiety medications are not recommended for temporary anxiety, such as that before an operation. Contrastingly, relaxation therapy is a simple, easy, and effective non-pharmacological treatment to relieve anxiety (14) and can be implemented with high compliance. Therefore, relaxation therapy can be used to alleviate anxiety and reduce the incidence of intraoperative hypoxia. This study aimed to evaluate whether relaxation therapy can reduce the incidence of intraoperative hypoxia among patients with pre-operative anxiety before undergoing painless artificial abortion. The results of this study can guide in the further improvements in perioperative safety and comfort.

## Methods

The study protocol was approved by the Ethics Committee of Shanghai Tenth People's Hospital (approval number: SHSY-IEC-4.1/20-47/01). The study was registered with the Chinese Clinical Trial Registry (registration number: ChiCTR2000032109) on April 20, 2020 at https://www.chictr.org.cn/index.aspx. This randomized controlled trial was conducted between September 2020 and January 2021. The participants included 280 anxious female patients who underwent painless artificial abortion in the outpatient department of Shanghai Tenth People's Hospital (Shanghai, China). All patients signed an informed consent form before surgery. Inclusion criteria were as follows: (1) Spielberger State-Trait Anxiety Inventory (S-STAI) score >45.5, (2) age 18–45 years, (3) body mass index 18.5–25.0 kg/m^2^, (4) 6–9 weeks of pregnancy, (5) American Society of Anaesthesiologists (ASA) physical status class I/II, (6) Mallampati airway classification class I/II, and (7) no coagulation dysfunction. Exclusion criteria were the following: (1) previous motion sickness, hypertension, heart disease, asthma, epilepsy, Parkinson's disease, depression, and other diseases, (2) history of drug allergy, and (3) routine blood oxygen saturation (SpO_2_) ≤ 98%.

Data were collected 1 h before surgery through a questionnaire. The participants were asked about their general information, anxiety scores using the S-STAI, and informed consent. They were categorized into a control group and an experimental group through a random number generated by a computer. Patients in the experimental group received pre-operative relaxation therapy. The surgeon and the anaesthesiologist did not intervene in the assignment of numbers and were blinded to the groups.

Half an hour before the operation, patients in the experimental group received audio therapy and progressive relaxation training in a separate room in the presence of an observer. The patients were instructed to close their eyes in an open and quiet place, to keep quiet, and to perform 10 min of progressive muscle relaxation training according to the audio instructions (15). Voluntary and orderly muscle relaxation and contraction were required until all muscle groups were relaxed. Progressive muscle relaxation started with the hands, followed by the arms, shoulders, chest, legs, and feet to exercise all muscle groups. Six different types of music were played in a loop, such as the piano, harp, orchestra, jazz, Chinese orchestra, and synthesizer. The music rhythm ranged from 60 to 80 beats per min, the pitch was low, melody lines were smooth, and the volume and rhythm did not change significantly (16, 17).

The anaesthesiologist conducted routine monitoring by measuring the blood pressure, heart rate, SpO_2_, and baseline bispectral index scores before the operation (T0) and 1 min (T1) and 5 min (T5) before the beginning of the operation. Oxygen at 6 L min^−1^ (37°C, oxygen concentration 100%) was administered to the patient through a face mask. The patient then received an intravenous bolus of 0.01 mg kg^−1^ butorphanol tartrate (Nuoyang) for analgesia and 2 mg kg^−1^ of propofol for anesthesia induction. The operation started after the disappearance of ciliary reflex. In case of intraoperative body movement, 0.5 mg kg^−1^ of propofol was administered. Simultaneously, we recorded the number of body movements and the number of times additional propofol was required. The aim was to maintain the bispectral index score between 50 and 70. Hypoxia events were recorded when the patients' SpO_2_ was <95% and lasted more than 15 s (18). Sedation-related adverse events were defined as systolic blood pressure <90 mmHg, heart rate <50 beats min^−1^, mild hypoxia (75% ≤ SpO_2_ <90%, with a duration <60 s), or severe hypoxia (SpO_2_ <75% or 75% ≤ SpO_2_ <90%, with a duration ≥60 s). In case of adverse effects, norepinephrine (20–40 μg) was administered to patients with hypotension, and atropine (0.25–0.50 mg) to patients with bradycardia. In patients with hypoxia and severe hypoxia, the jaw was lifted to open the airway and increase oxygen flow. Thereafter, mask ventilation was used to treat hypoxia. If hypoxia could not be treated by mask ventilation, endotracheal intubation was performed. Patients with sedation-related adverse events were excluded from the study. All sedation procedures in this study were performed by an anaesthesiologist.

Based on the results of our previous clinical trials, we determined that the cut-off S-STAI score should be >45.5 points. There is an increased risk of intraoperative hypoxia when the score is >45.5 points. The primary outcome indicator of this study was the incidence of hypoxia, and the secondary outcome indicators were the dosage of propofol, number of times additional propofol was required, incidence of body movement, and change in heart rate and mean arterial pressure.

### Sample Size Calculation

The calculation of the sample size was based on the pre-experimental prevalence rate of 23.3%. The sample size ratio of the control and experimental groups was 1:1; the number of cases and controls was calculated at 110 cases each. We selected 280 patients to account for the 20% dropout rate.

### Statistical Analysis

SPSS version 20.0 software was used for data analysis. Continuous variables are represented as mean and standard deviation and categorical variables are represented as frequency and proportion. Continuous variables were analyzed by two-sample *t*-test and the categorical variables were analyzed by independent-sample *t*-test and Fisher test. A value of *p* < 0.05 was considered statistically significant.

## Results

**Figure 1:**
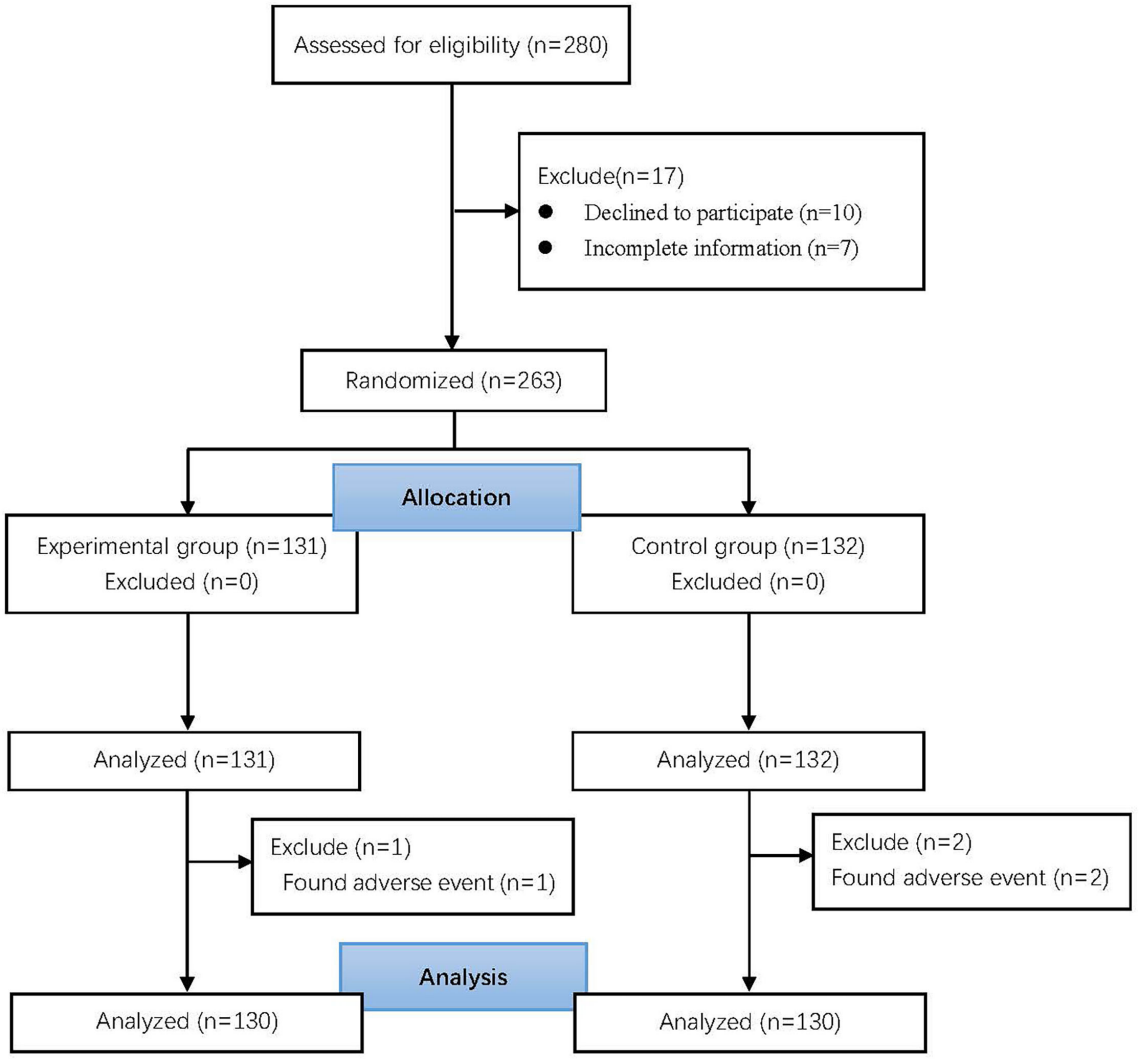
Flowchart of selection process.

Of 1,985 patients, 280 were recruited based on anxiety scores. Among them, 17 were excluded (10 patients refused to participate and 7 did not complete the pre-operative survey). The remaining 263 patients were randomly categorized into two groups. Three patients (2 in the control group and 1 in the experimental group) had adverse reactions and were eventually excluded. Finally, 260 patients were analyzed (Figure 1).

There was no significant difference between the two groups in terms of age, height, body mass index, heart rate, mean arterial pressure, the number of days since last menstruation, smoking and drinking habits, and S-STAI scores (Table 1).

**Table 1:** Patient characteristics.

**Factors**	**Experimental group (*n* = 130)**	**Control group** **(*n* = 130)**	***P***
Age (years)	30.96 ± 6.11	31.05 ± 5.98	0.902
Height (cm)	162.05 ± 4.31	161.68 ± 4.51	0.500
Body mass index (kg/m^2^)	21.10 ± 2.66	21.49 ± 2.50	0.221
Pre-operative heart rate (times/min)	83.22 ± 14.88	87.51 ± 16.71	0.262
Pre-operative mean arterial pressure (mmHg)	88.63 ± 10.12	85.61 ± 10.20	0.575
Pre-operative oxygen saturation (%)	100	100	/
Menopausal days (days)	51.45 ± 5.70	50.45 ± 6.04	0.174
Smoking (%)			
Yes	13.1	8.5	
No	86.9	91.5	0.230
Drinking (%)			
Yes	4.6	5.4	
No	95.4	94.6	0.776
S-STAI score (points)	57.27 ± 3.56	56.88 ± 2.91	0.331

*Values are presented as mean ± standard deviation or n (%).*

*S-STAI, Spielberger State-Trait Anxiety Inventory*.

**Figure 2:**
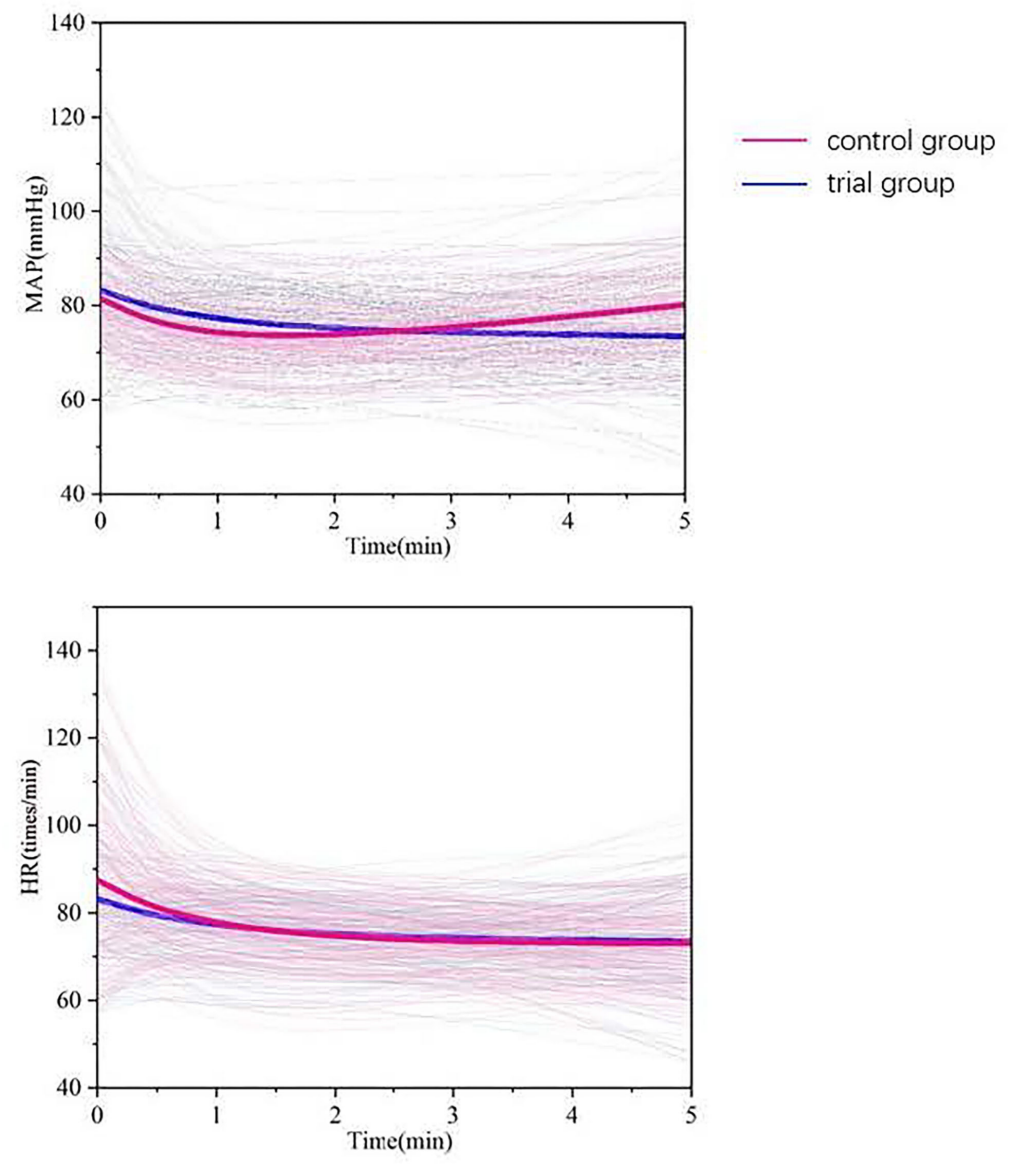
Average values of the hemodynamic variables studied (thick line) and individual patient data (thin line) : the red line represents the control group, and the blue line represents the trial group.

In the experimental group, the incidence of hypoxia dropped from 30 to 12.3%, which was a 59% reduction, after pre-operative muscle relaxation. Moreover, the consumption of propofol in the experimental group during induction of anesthesia was significantly less than that in the control group (13.91 ± 2.26 vs. 14.58 ± 3.07, *p* = 0.046). The number of times additional propofol was required in the experimental group was significantly less than that in the control group (1.08 ± 0.88 vs. 1.26 ± 1.06, *p* = 0.047; Table 2). The changes in heart rate and mean arterial pressure over time after anesthesia induction are shown in Figure 2, and the hemodynamic data are shown in Tables 3, 4. Compared with baseline (T0), the heart rates of both the experimental and control groups were significantly reduced 5 min (T5) after the induction of anesthesia.

**Table 2:** Perioperative indicators.

**Factors**	**Experimental group (*n* = 130)**	**Control group** **(*n* = 130)**	***P***
The incidence of hypoxia (%)	12.30%	30%	<0.001
Propofol dosage (mg)	13.91 ± 2.26	14.58 ± 3.07	0.046
Propofol bolus (times)	1.08 ± 0.88	1.26 ± 1.06	0.047
Incidence of body movement (%)			
Yes	50	53.8	0.535
No	50	46.2	

*Values are presented as mean ± standard deviation or %*.

**Table 3:** Intraoperative haemodynamic data.

**Factors**	**Experimental group (*n* = 130)**	**Control group** **(*n* = 130)**	***P***
Changes in heart rate (times/min; T5–T0)	−9.68 ± 11.46	−14.45 ± 14.19	<0.001
One min mean arterial pressure changes (mmHg; T1–T0)	−12.07 ± 11.19	−12.44 ± 9.45	0.745
Five min mean arterial pressure changes (mmHg; T5–T0)	−1.63 ± 12.04	−1.42 ± 10.82	0.867

*Values are presented as mean ± standard deviation*.

**Table 4:** Intraoperative haemodynamic data.

		**T0**	**T5**	**T5–T0**
Heart rate (times/min)	Experimental group	83.22 ± 14.88	73.54 ± 10.46	−9.68 ± 11.46
	Control group	87.51 ± 16.71	73.05 ± 10.22	−14.45 ± 14.21
	*P*	0.262	0.662	<0.001
Mean arterial pressure	Experimental group	88.63 ± 10.12	86.99 ± 11.18	−1.63 ± 12.04
(mmHg)	Control group	85.61 ± 10.20	84.19 ± 11.01	−1.42 ± 10.84
	*P*	0.575	0.954	0.867

*Values are presented as mean ± standard deviation*.

## Discussion

Pre-operative anxiety is a potential conscious response of the human brain. Anxiety is magnified when people feel threatened with externalities. Mild anxiety can improve a person's ability to handle stress, while excessive anxiety may lead to hemodynamic changes during the operation, and this can seriously affect the patient's perioperative safety.

People usually have a stress response due to pre-operative anxiety (19) that leads to increased sympathetic nerve activity and increased release of catecholamines from the adrenal medulla (20). This results in increased blood pressure and heart rate, accelerated breathing, other adrenergic responses, and pituitary–adrenal cortex reactions (21, 22). The administration of anti-anxiety drugs for pre-operative anxiety is not advisable because it may cause adverse reactions such as excessive sedation, hypotension, allergies, vomiting, and shock (23, 24). These complications increase costs and side effects. However, relaxation therapy provides a good anti-stress effect that can reduce sympathetic nerve activity. Consequently, there is a reduction in the respiratory rate, heart rate, and blood pressure, and an increase in the feeling of wellness. The relaxation therapy employed in this study included music therapy and progressive muscle training. Both relaxation methods can reduce pre-operative anxiety (25, 26) and have a good synergistic effect.

The main purpose of this study was to explore the effect of relaxation therapy on hypoxia in patients with anxiety before painless abortion. We defined hypoxia as SpO_2_ <95% and lasting more than 15 s. The monitoring of SpO_2_ was delayed because apnea and altered respiration frequently precede hypoxemia with a significant time lag of up to 2 min (27, 28). According to the obtained results, the incidence of hypoxia in anxious patients after relaxation therapy was reduced by 59%. Further, the number of times additional propofol was required and total consumption of propofol were significantly reduced. Considering the respiratory depression effect of propofol, we believe that the lower incidence of hypoxia in the experimental group may be the combined effect of using lesser amount of propofol during surgery.

In this experiment, six different types of music were played in a loop. Music can heal the body and mind (29). Although personal preferences for music vary, music can play a direct physiological role through the autonomic nervous system (30). Furthermore, unlike other relaxation methods such as massage and yoga, it is a low-cost intervention. Since the early 1990s, music therapy has been used to aid in sleeping and to reduce anxiety related to surgery (31). Now, music therapy is used in different fields. Listening to music can significantly reduce anxiety (*p* = 0.003) in cardiac catheterization. In a previous study, the music group exhibited decreased heart rate and increased skin temperature (*p* < 0.001) (16). Music therapy is effective in reducing anxiety in critically ill patients (32). Additionally, music therapy is a safe and low-cost intervention that can relieve pain and anxiety associated with surgery (33).

Progressive relaxation training helps patients relax all muscle groups. All relaxation processes ensured rhythmic breathing, muscle tension reduction, and increased patient awareness. The method is simple and easy to implement, without external intervention. As an auxiliary method, progressive muscle relaxation can reduce patient anxiety. For example, it can help improve sleep quality in patients with coronavirus disease 2019 (34). In patients undergoing gastrointestinal surgery, progressive relaxation training can increase their pain threshold and reduce their stress and anxiety (35).

In our previous study, we found that anxiety is the main risk factor of hypoxia. We assumed that relaxation therapy would reduce the rate of hypoxia of anxious patients. The results of this study showed that the incidence of hypoxia in the experimental group was 12.3% and in the control group was 30% (*p* < 0.001).

The current study also showed that relaxation therapy can effectively reduce the consumption of propofol. The comparison of the average of the two groups showed that the dose of propofol in the control group was higher than that in the experimental group. Therefore, for painless abortion surgery, anxious patients who used relaxation therapy required less amount of propofol. Higher levels of anxiety among patients increased their needed amount of propofol intraoperatively. These results were similar to the results of Li Ruiyun (36). Music has a sedative effect on patients. It can change their physiological parameters and reduce pain and discomfort, thereby reducing the required analgesic dose (37). Our results show that relaxation therapy can reduce the dosage of intraoperative propofol by relieving pre-operative anxiety, thus reducing the incidence of intraoperative hypoxia.

Like most studies, this study has some limitations that may affect the experimental results. First, the sample size of the experiment is small. Not all anxious patients were included in the group due to the S-STAI score cut-off at 45.5 points. Second, this is a randomized single-blind experiment. Since the experimental group required relaxation therapy, a double-blind control could not be achieved. Third, there are differences in the surgical techniques of different gynecological surgeons, even if uniform training is carried out. Fourth, due to the time constraints, we did not record the S-STAI scores after relaxation therapy. Furthermore, participants were unable to individually choose their favorite music during the experiment. This may reduce the effect of music therapy.

## Conclusion

This study focused on the effect of relaxation therapy on hypoxia caused by intravenous propofol anesthesia in patients with pre-operative anxiety. We found that relaxation therapy can reduce the occurrence of hypoxia in anxious patients, and it may be the combined effect caused by the use of less dosage of propofol during the operation.

## Implication Statement

This study focused on the effect of relaxation therapy on hypoxia caused by intravenous anesthesia with propofol in patients with pre-operative anxiety. We found that relaxation therapy can reduce the occurrence of hypoxia in anxious patients and improve perioperative safety.

## Data Availability Statement

The raw data supporting the conclusions of this article will be made available by the authors, without undue reservation.

## Ethics Statement

The studies involving human participants were reviewed and approved by Ethics Committee of Shanghai Tenth People's Hospital (approval number: SHSY-IEC-4.1/20-47/01). The patients/participants provided their written informed consent to participate in this study.

## Author Contributions

YF and QJ: data collection. YF, QJ, and CL: data analysis. YF and CL: writing. QJ, SC, XS, HZ, LT, and CL: study design. YF: patient recruitment. YF and QJ: contributed equally to this work. All authors: revision. All authors contributed to the article and approved the submitted version.

## Funding

This study was supported by the Natural Science Foundation of Shanghai [grant number: 20ZR1442900) and Shanghai Fourth People's Hospital, School of Medicine, Tongji University (grant numbers: sykyqd01901 and SY-XKZT-2021-2001).

## Conflict of Interest

The authors declare that the research was conducted in the absence of any commercial or financial relationships that could be construed as a potential conflict of interest.

## Publisher's Note

All claims expressed in this article are solely those of the authors and do not necessarily represent those of their affiliated organizations, or those of the publisher, the editors and the reviewers. Any product that may be evaluated in this article, or claim that may be made by its manufacturer, is not guaranteed or endorsed by the publisher.
